# Effect of sacubitril/valsartan on sinus rhythm maintenance after catheter ablation in patients with persistent atrial fibrillation without reduced ejection fraction heart failure: a study protocol for a multi-center, open-label, randomized, controlled, superiority clinical trial

**DOI:** 10.3389/fcvm.2024.1303540

**Published:** 2024-01-29

**Authors:** Ruowu Qiu, Qingqing Ni, Muli Wu, Zhongbo Xiao, Jiaxin Xiao, Weizhao Lin, Weipeng Huang, Yequn Chen, Chang Chen, Liekai Hong

**Affiliations:** ^1^ Cardiology Department, The First Affiliated Hospital of Shantou University Medical College, Shantou, Guangdong, China; ^2^ Shantou University Medical College, Shantou, Guangdong, China; ^3^ Clinical Research Center, The First Affiliated Hospital of Shantou University Medical College (SUMC), Shantou, Guangdong, China; ^4^ Cardiology Department, The Second Affiliated Hospital of Shantou University Medical College, Shantou, Guangdong, China; ^5^ Cardiology Department, Jieyang People’s Hospital, Jieyang, Guangdong, China

**Keywords:** sacubitril/valsartan, persistent atrial fibrillation, catheter ablation, sinus rhythm maintenance, major adverse cardiovascular events

## Abstract

**Introduction:**

A high recurrence rate of atrial fibrillation was monitored after catheter ablation for persistent atrial fibrillation. Sacubitril/valsartan can improve outcomes for patients with heart failure and ventricular tachycardia, but few studies examined whether it can reduce recurrence or improve cardiovascular outcomes in patients with persistent atrial fibrillation after catheter ablation. In this study, we will assess the effect of sacubitril/valsartan on sinus rhythm maintenance and incidence of major adverse cardiovascular events (MACE) in patients with persistent atrial fibrillation after catheter ablation through a randomized controlled trial (RCT).

**Methods:**

This is a multi-center, randomized, controlled, open-label, superiority clinical trial involving 462 patients without reduced ejection fraction heart failure after catheter ablation of persistent atrial fibrillation. Patients will be randomized to (1) receive the standard treatment strategy plus sacubitril/valsartan titration, or (2) receive the standard treatment strategy without taking sacubitril/valsartan. The primary outcome will be sinus rhythm maintenance rate over 12 months, monitored by random electrocardiogram and 24-h Holter electrocardiogram.

**Discussion:**

This study is designed to evaluate the effect of sacubitril/valsartan on sinus rhythm maintenance and incidence of major adverse cardiovascular events (MACE) in patients with persistent atrial fibrillation after catheter ablation. The results will evaluate sacubitril/valsartan as a novel treatment for improving prognosis and a complement to conventional drug therapy.

**Trial Registration:**

Registered with Chinese Clinical Trials Registry on 27 August 2022, identifier: ChiCTR2200062995.

## Introduction

Atrial fibrillation (AF) now has become a common clinical arrhythmia and refers to the loss of ordered electrical activity in the atria. In China, the available evidence infers that the prevalence of AF in China is 2.32% among people over 65 years old (1–4). The incidence of AF has increased continuously over the past decades, and the rates of hospitalization and fatality are also increasing (5). Based on the duration of AF, AF can be divided into five categories: first diagnosed AF (AF not previously diagnosed), paroxysmal AF (duration < 7 days), persistent AF (duration > 7 days), long-standing persistent AF (duration > 1 year), and permanent AF (diagnosed as AF, patient and doctor have reached an agreement and will no longer attempt to restore sinus rhythm). The sustained onset of atrial fibrillation significantly increases the risk of cardiovascular and cerebrovascular accidents, affecting quality of life and survival of patients. A cohort study showed that the proportion of HF patients with AF is as high as 24.4%, and HF can make further efforts to induce AF (6). Chronic heart failure and persistent atrial fibrillation can promote each other, thus entering a vicious cycle (7, 8).

The therapy of atrial fibrillation has become an increasingly great challenge in the cardiovascular field. Catheter ablation has been confirmed to be an effective way to treat atrial fibrillation, and it has become the most recommended treatment option for symptomatic atrial fibrillation (9). Cochrane systematic reviews have also shown that ablation of persistent AF had a greater advantage in prognosis than anti-arrhythmic drug treatment in eliminating atrial arrhythmias, reducing the need for cardioversion and the risk of hospitalization for cardiac reasons (10). However, the recurrence rate of AF still remained high in patients after catheter ablation, with incidence of early postoperative recurrence (3–12 months) being 25%–40% (11). The CABANA clinical study showed a recurrence rate of 49.9% in the AF ablation group during a long period of follow-up (12). After undergoing catheter ablation, the recurrence rate of persistent AF is higher compared to paroxysmal AF (11). Reducing the recurrence of atrial fibrillation can effectively improve the prognosis of catheter ablation.

Sacubitril/valsartan is the first angiotensin receptor-neprilysin inhibitor (ARNI) with a dual-target inhibitory effect. Studies have shown that AF patients with HF, treated with ARNI, tended to reduce the risk of hospitalization, reversal of cardiac remodeling, and corrected cardiac function after catheter ablation of AF (13–15). However, there is a lack of randomized controlled trials (RCT) to show a therapeutic effect of ARNI in patients with persistent AF after catheter ablation. We will evaluate the effect of ARNI on sinus rhythm maintenance rate and incidence of MACE in patients with persistent AF after catheter ablation in this study through a randomized controlled trial.

## Methods and analysis

### Study design

This prospective trial is a multi-center, randomized, controlled, open-label, superiority trial with a 1:1 ratio of trial and controls. A total of 462 patients with persistent atrial fibrillation after catheter ablation will be recruited in three centers (the First Affiliated Hospital of Shantou University Medical College, Second Affiliated Hospital of Shantou University Medical College, and Jieyang People's Hospital) from August 2022 through August 2025. This protocol is designed according to the Standard Protocol Items: Recommendations for Interventional Trials (SPIRIT) 2013 Statement (16).

### Study hypothesis and objectives

The overall expectation is that patients taking sacubitril/valsartan with persistent atrial fibrillation after catheter ablation will have better sinus rhythm maintenance and cardiovascular outcomes within 12 months than those not taking. This prediction will be tested with the following three objectives. The primary objective will be to evaluate the efficacy of sacubitril/valsartan on sinus rhythm maintenance after ablation of persistent AF patients in this trial. Whether sacubitril/valsartan decreases the incidence of MACE and improves structural indices of the atrium in patients with AF is the secondary objective of this study. The third objective will be to determine the efficacy of sacubitril/valsartan on re-hospitalization rates for cardiovascular causes.

### Population

Patients will be recruited at The First Affiliated Hospital of Shantou University Medical College, The Second Affiliated Hospital of Shantou University Medical College, and Jieyang People's Hospital, three medical centers in Guangdong province. A well-trained research team composed of clinicians familiar with the diagnosis and treatment of atrial fibrillation was set up to screen patients who may meet the research criteria.

### Ethics issues

Ethical approval was obtained before the start of this study from the Committee of Ethics of the First Affiliated Hospital of Shantou University Medical College (Number: B-2022-170-XZ1).

### Inclusion criteria

(1) Adult (age ≥ 18), male or female
(2) Physical condition allows oral medication
(3) Patients without HFrEF (heart failure with LVEF ≤ 40%)
(4) Patients who have a clinical diagnosis of persistent AF (AF lasting >7 days, Holter ECG >24 h will be completed before procedure) and have undergone catheter ablation (successful conversion of rhythm and immediately complete electrocardiogram after procedure)
(5) Understand the purpose of the trial and agree to all the study steps, voluntarily sign the informed consents, and be willing to undergo follow-up.

### Exclusion criteria

(1) Pregnant and lactating women
(2) Patients with previous ablations (percutaneous or surgical)
(3) Acute coronary syndrome (including myocardial infarction), acute stroke, or emergency coronary intervention within 3 months prior to enrollment
(4) Heart failure: a clinical diagnosis of heart failure, and transthoracic heart color ultrasound indicates that the left ventricular ejection fraction is 40% or less
(5) Patients with acute infectious diseases, important organ injuries, malignant tumors
(6) Severe liver insufficiency (Child-Pugh level C) and severe renal insufficiency (eGFR <30 ml/min/L.73 m^2^)
(7) The average blood pressure of the three postoperative measurements is lower than 90/60 mmHg
(8) Abnormal coagulation mechanism and immune function
(9) The subject is currently taking part in another clinical study
(10) The investigator considers that the subject is unable to follow the protocol requirements or for any reason considers that the subject is not eligible for the study
(11) Allergic to the active ingredient of sacubitril/valsartan or any excipients.

### Withdrawal criteria

Patients can voluntarily withdraw from this clinical trial at any time. Patients who experience the following conditions will also withdraw from the trial:

(1) Serious adverse events (AEs) occurred throughout the entire trial process
(2) Unable to continue cooperating with clinical examinations and follow-up due to unexpected reasons.

### Informed consent

Throughout the entire trial enrollment process, patients who have been assessed as eligible for enrollment will be introduced to the research content and specific process by trained researchers, ensuring that patients fully understand the benefits and potential risks of the trial. At the same time, patients will be informed that whether they participate or not will not affect their normal treatment and all participants' personal information will always be kept confidential. Patients will have 24 h to consider and make decisions. All participating patients must sign the informed consent.

### Randomization and allocation

Patients who meet the inclusion criteria, successfully converted to sinus rhythm and were confirmed by electrocardiogram immediately after ablation will be enrolled. Randomization will be performed after the ablation. After eligible patients sign the informed consent, baseline information will be collected, including basic information, medical history, preoperative medication history, electrocardiogram and laboratory results. Researchers will randomly allocate eligible patients at a 1:1 ratio to receive sacubitril/valsartan or not. This trial will be randomized using a dynamic stratified block group randomization method. The R package blockrand (R version 4.1.2) was used to generate a randomization table with a specified sample size, and the block sizes were randomized among 4, 6, and 8 to form the randomization pool for this trial. The trial was stratified by research centers, each of which was assigned random numbers for a block unit by “first-come, first-served”, and the random number for the next block was available in the randomization pool only when the random number for that block was used up. The process of generating the randomization table and assigning randomized numbers for this study was carried out by an independent researcher who was not involved in recruitment and subsequent interventions. After a subject had consented to participate in the trial, the recruiting physician queried the randomization number from the investigator in charge of randomization, who provided the patient's treatment allocation plan based on the participant's center and order of inclusion, as well as marked the grouping on the randomization table.

### Interventions

**Figure 1:**
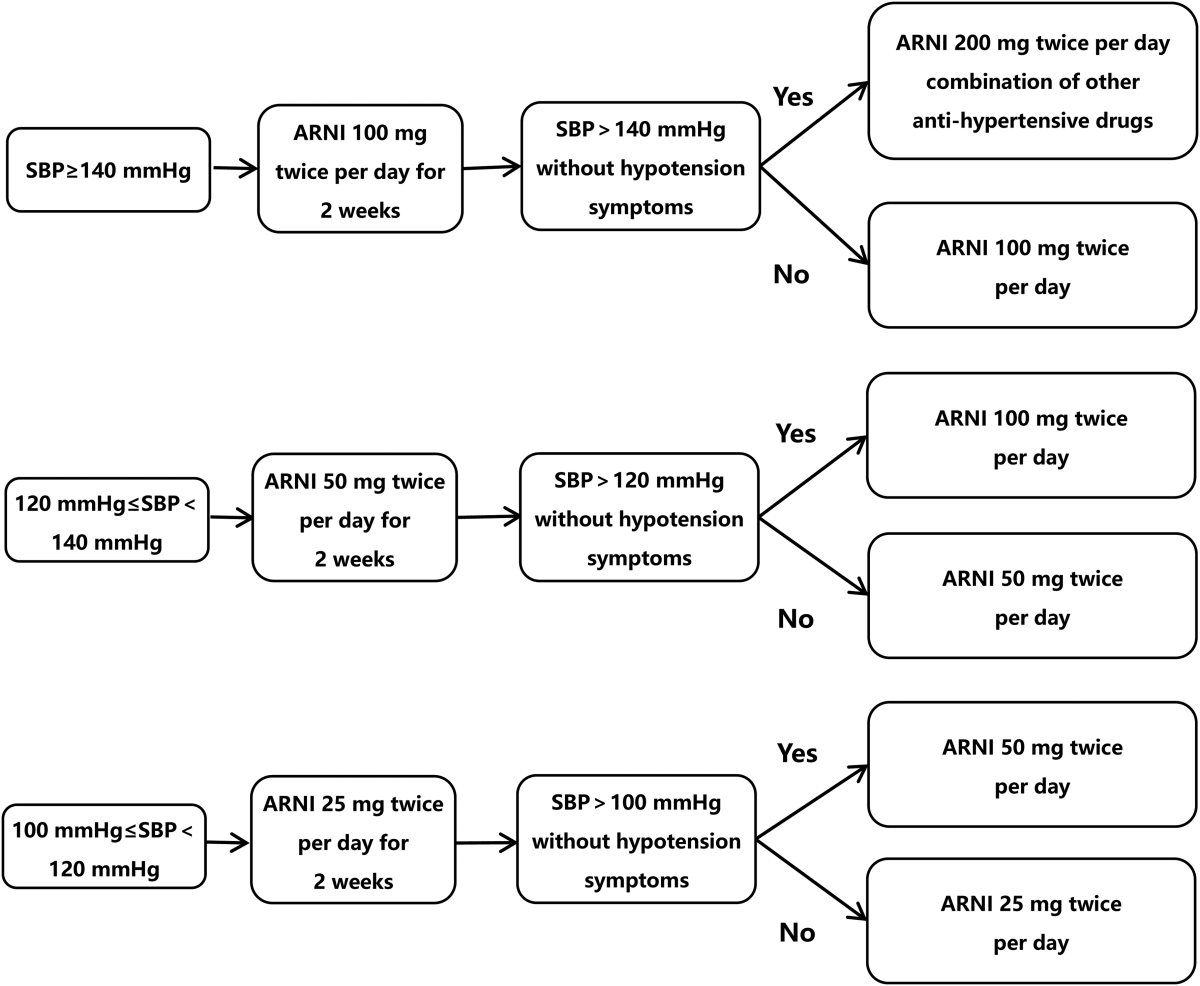
Drug dose titration.

For all patients enrolled, if they are taking ACEI drugs, they will be given the trial drug starting 36 h after discontinuation of ACEI drugs. The control group will be given conventional perioperative medications according to guidelines of atrial fibrillation, and the trial group will receive conventional perioperative drug therapy plus sacubitril/valsartan (Beijing Novartis Pharmaceutical Company, J20171054). The test drug will be titrated according to Figure 1. The target maintenance dose is 100 mg twice per day. Based on the patient tolerance, drug dosage will be doubled every 2 weeks. If the patient cannot tolerate the maximum dose, the dose will be reduced to the maximum dose tolerated by the patient. In order to increase patient medication compliance, patients will be reminded to take drugs on time by WeChat. When patients change the dose, patients will be required to bring the packaging box of the drugs. Blood pressure, blood potassium and renal function examination will be performed at each follow-up period. If hypotension or symptomatic hypotension occurs, the dose will be adjusted.

### Type of ablation

All patients will underwent pulmonary vein isolation (PVI) using radiofrequency energy and a three-dimensional electroanatomical mapping system (CARTO, Biosense Webster). Additional ablation lines (roof line of the LA, mitral isthmus line, or cavo-tricuspid isthmus line) or ablation of complex fractioned electrograms would not be performed during procedure for enrolling patients.. The endpoint for complex fractionated atrial electrogram ablation is a complete abatement of potentials at these sites.

### Conventional perioperative medications

Patients will be treated by standard treatment according to current guidelines (11). They will be on an uninterrupted anticoagulation regimen during the perioperative period. Uninterrupted oral anticoagulation will be taken based on stroke/thromboembolic risk during the perioperative period of AF catheter ablation for enrolling patients (9). Patients can use antiarrhythmic drugs such as Class III drugs (amiodarone) when appropriate based on the judgment of the responsible physician during the 3-month blanking period.

### Blinding

This trial is designed as an open label. Trial participants and care givers will not be blinded to the group assignment while the investigator or other relevant personnel will not be made aware of the information about the treatment subgroup to which they are assigned until the order of enrollment/randomization of the subjects has been confirmed. Data analysts and outcome evaluator will remain blinded to minimize trial bias.

### Outcome measurements

#### Primary outcome

The primary endpoint of the study will be the sinus rhythm maintenance rate within 12 months determined by at least the 24-hour Holter electrocardiogram. Patients will be given the Holter electrocardiogram at 3, 6 months and 12 months.

#### Secondary outcome

The secondary endpoint will be the sinus rhythm maintenance rate at 3 and 6 months and incidence of MACE at 3, 6, and 12 months. Cardiovascular re-hospitalization at 3, 6, and 12 months will be aggregated in HFpEF patients with AF and patients with AF and otherwise normal cardiac function. Changes in cardiac structural function indicators will be observed at 3, 6, and 12 months. Echocardiographic parameters such as LAD, LAVi, LAEF will be recorded or calculated.

#### Safety outcome

This study will also analyze the safety profile focusing on the incidence of adverse events at months 3, 6, and 12. Blood pressure will be measured and potassium and renal function will be tested during follow-up period. The main adverse events will include hypotension, hyperkalemia and renal function impairment that cannot be improved by dose reduction. Hyperkalemia (blood potassium > 5.5 mmol/L), hypotension (SBP < 90 mmHg or DBP < 60 mmHg), symptomatic hypotension (SBP ≥ 90 mmHg and dizziness, nausea, fatigue, syncope), and creatinine increase more than 50% will be considered as adverse reactions.

### Definition

This study differentiate different cardiovascular causes for re-hospitalization into cardioversion, repeat ablation, heart failure, acute coronary syndrome, stroke or transient ischemic attack, or other cardiovascular-related diseases.

MACE are defined as heart failure, myocardial infarction, stroke, malignant arrhythmia and cardiac death. The specific definitions are as follows:

(1) Definition of heart failure: patients who had a history of HF-plausible symptoms, physical examination changes, electrocardiogram changes, cardiac ultrasound changes, and elevated serological markers of heart failure (NT-proBNP > 125 pg/ml or BNP ≥ 35 pg/ml) will be clinically diagnosed as heart failure according to the 2022 AHA/ACC/HFSA Guidelines: Management of Heart Failure. Heart failure with reduced ejection fraction (HFrEF) is classified as an LVEF ≤ 40% (17).
(2) Definition of acute coronary syndrome: includes ST-segment elevation myocardial infarction (STEMI), non-ST-segment elevation myocardial infarction (NSTEMI), and unstable angina (UA) according to the guidelines for rapid diagnosis and treatment of acute coronary syndromes in emergency medicine (18).
(3) The diagnosis of malignant arrhythmia (MA) refers to the diagnostic criteria for malignant arrhythmia in the 2020 American Heart Association Guidelines for Resuscitation and Emergency Cardiovascular Care (19). MA includes ventricular arrhythmias (ventricular tachycardia and ventricular fibrillation) and grade II or III atrioventricular block. Each type of MA may occur alone or in combination with other types at the same time.

### Follow-up

**Figure 2:**
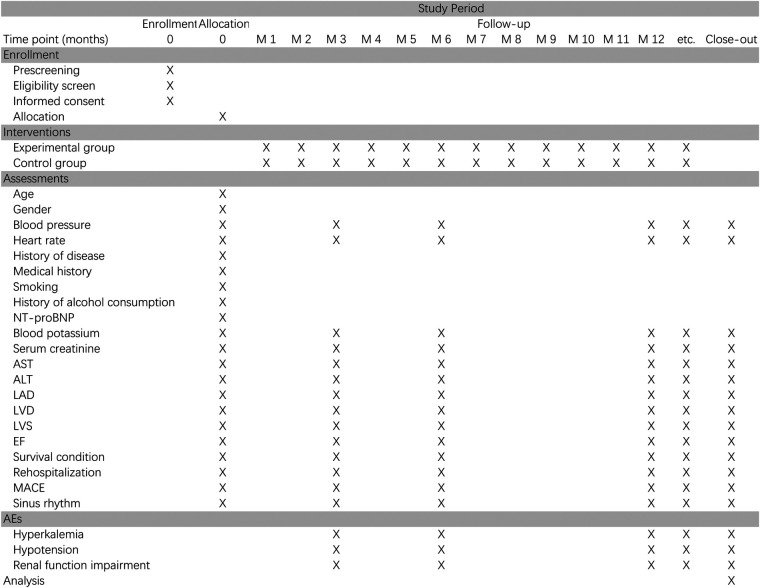
Schedule of enrollment, interventions and assessments for the trial.

After randomization, subjects will have follow-up after 3, 6 and 12 months, mainly in the outpatient clinic according to Figure 2.

(1) The survival status will be confirmed and rehospitalization and occurrence of MACE events will be requested by telephone
(2) Holter electrocardiogram (>24 h), transthoracic heart color ultrasound, blood test of blood potassium and kidney function will be performed in the outpatient clinic
(3) Adverse event assessment, the main adverse reactions assessed will be: hypotension, high blood potassium, impairment of renal function, as determined in the outpatient clinic
(4) Medication consumption in the past will be requested by telephone;
(5) Drug bottles will be collected, and the subject reminded of the importance of study medication compliance by the outpatient clinic.

### Sample size

The determination of the sample size in this study will be driven by the need of the above strategy to detect sacubitril/valsartan over conventional drugs.

The recurrence rate of atrial fibrillation after 12 months in patients with persistent AF is approximately 42% (15), and we expect a 20% decrease in the incidence of primary endpoint events taking ARNI after catheter ablation in patients with persistent AF. Under the assumption based on the exponential distribution, the risk ratio is 0.46.

Based on the above assumptions, the expected enrollment time is 24 months, without considering the mid-term analysis, and the minimum follow-up time is 12 months. Assuming a 20% missing visit in each of the control and trial groups, the study requires 450 participants (225 trial group, 225 control group) to obtain 90% difference at a significance level of *a* = 0.05 (unilateral). The sample size is calculated by PASS15.0 software. The interim analyses is set up by the O'Brien-Fleming method to control the overall Type I error below 0.05. The final sample size will be 462 cases (231 trial group, 231 control group).

### Data collection and management

Blank case report forms (CRFs) that has been ethically approved will be used to record data for all participants. Each enrolled patient corresponds to a complete CRF, and unrecorded data will be labeled and justified. The CRFs will be entered into the scientific research platform of the First Affiliated Hospital of Shantou University Medical School in a double-entry manner by researchers. Researchers are responsible for the completeness and accuracy of the records. A CRF will include:

(1) Medical history or medical examination data
(2) Informed consent of the subjects and the randomization number of this trial
(3) Time of each follow-up visit and signed notes
(4) Reported adverse events and their resolution or absence, including documentation such as hospitalization records, discharge summary, ECG, etc
(5) Record of the medication use of the test subjects during the trial

### Statistical and analytical plans

The statistical analysis will be along with the intention-to-treat (ITT) principles. The ITT population will include patients who meet the criteria, are randomized, and take at least one dose of drugs after being enrolled. For statistical description, continuous variables are described by using mean and standard deviation if they satisfy a normal distribution, skewed distributions are described by medians and quartiles, while categorical variables will be presented as frequency or percentage in each category. For the baselines, the t-test is used to compare the differences for quantitative variable if the data meets the normality test, otherwise, the data will be transformed or rank sum test. Categorical variables such as disease history and smoking history can be tested using chi-square test or rank sum test. For outcomes such as 239sinus rhythm maintenance rate, all-cause death, re-hospitalization rate for cardiovascular causes and MACE, Kaplan-Meier curves will be used to characterize how the probability of an endpoint event changes with survival time, and comparisons between survival curves will be carried out using log-rank tests. We used cox proportional hazards models to estimate hazard ratios and 95% confidence intervals. Cardiac structural function indicators will be described by mean value (±SD) or median and quartiles, and the sphericity of the covariance array will be tested by the Mauchly method. If the data satisfies the sphericity of the covariance array, the analysis of variance with repeated measures data will be used for group comparisons of each indicator. If it is not satisfied, the Greenhouse-Geisser method will be used for comparison of differences. Missing observations will be accounted for using the predictive mean matching (PMM) method. If the missing data is numerical, it will be filled by predictive mean matching; meanwhile if the missing data is non-numerical, logistic regression and discriminant functions will be used to fill it. The *p*-value threshold is 0.05(*p* < 0.05), and when it is below this threshold, indicating that the difference is statistical significant. SPSS statistical software (SPSS Inc, Chicago, IL, USA, version 23) will be used to analyse data.

Interim analyses of key safety and endpoint metrics will be conducted periodically during the course of the trial. Two interim analyses will be conducted when 33% and 67% of the total number of planned primary endpoint events have been reached. Interim analyses will be performed using the O'Brien-Fleming method to maintain an overall significance level of 0.05. The significance level alpha for the two interim and final analyses is 0.0015, 0.0172, and 0.0313, respectively. The independent Data Monitoring Committee will review the safety and efficacy of the trial based on interim analysis results.

### Monitoring

The trial will be conducted following the approved protocol. An independent committee of data and safety monitoring has been established to oversee the trial. Five chairpersons will be set up in the committee composed of independent researchers, while ensuring that committee members are not involved in the trial operation and have no conflicts of interest with this study. The data and safety monitoring committee will review and supervise the reliability and completeness of research data on the basis of ensuring the physical safety and data security of participants.

### Serious adverse events

Events that are fatal, life-threatening, disabling or incapacitating, or leading to prolonged hospital stay or malformation are defined as serious adverse events in this trial.

The patient blood pressure will be observed by follow-up, and blood samples will be tested for potassium and renal function. The main adverse events will include hypotension, hyperkalemia and renal function impairment that cannot be improved by dose reduction. Adverse reaction criteria: hyperkalemia (blood potassium > 5.5 mmol/L), hypotension (SBP < 90 mmHg or DBP < 60 mmHg), symptomatic hypotension (SBP ≥ 90 mmHg and dizziness, nausea, fatigue, syncope), and creatinine increase more than 50% will be considered as adverse events.

A detailed report will be submitted to the Medicines and Healthcare Products Regulatory Agency in a timely manner when unexpected serious adverse events occur. All patients participating in the trial will receive insurance, including additional healthcare, compensation, or damages. All adverse events which may possibly related to the trial will be recorded in the CRFs.

### Auditing and amendment

During the course of the study, representatives of the ethics committee will conduct reviews in accordance with national regulations. If there is a need to make changes to the protocol, an application will be submitted in advance to the ethics committee and regulatory agencies for approval, then recorded in the form of an amendment to the protocol.

### Public involvement

There was no public or patient involvement in the conceptualization of the study. The research results will be analyzed and be part of written article for publication.

## Discussion

This is a randomized, controlled, open-label, superiority clinical trial designed to explore the efficacy of sacubitril/valsartan on sinus rhythm maintenance in patients with persistent AF after catheter ablation. As a common cardiac arrhythmia worldwide, atrial fibrillation is closely related to various adverse outcomes and economic burden. It has been shown that persistent AF as an independent influencing factor is more significantly associated with increased mortality or all-cause hospitalization compared with paroxysmal AF or first diagnosed AF (20, 21). Cardiac electrical remodeling, due to cardiac reconstruction, left atrial volume enlargement, and atrial fibrosis, is important to the development and progression of atrial fibrillation. Especially in persistent atrial fibrillation patients, long-term arrhythmia further aggravate the electrical remodeling of the heart, while the chances of heart failure attacks are greatly increased. Otherwise, heart failure can cause atrial dilatation, which leads to cardiac damage and fibrosis (22, 23). All of these significantly contribute to the deterioration of AF patients. Catheter ablation of AF is an effective therapy and has become the first choice of treatment for symptomatic atrial fibrillation (9). However, patients with persistent AF after catheter ablation still remain a significant recurrence rate, which may be associated with heart reconstruction, partial myocardial fibrosis and structural changes that cannot be completely reversed by the procedure. Reducing the recurrence of atrial fibrillation can effectively improve the prognosis of catheter ablation.

Sacubitril/valsartan is the first angiotensin receptor-neprilysin inhibitor (ARNI) with a dual-target inhibitory effect. The mechanism of action is to reduce myocardial fibrosis and improve cardiac remodeling by inhibiting the RAAS system, modulating the natriuretic peptide system, and restoring neurohumoral homeostasis (24). The PARADIGM study and the PARAMOUNT study both showed good efficacy of ARNI (25, 26), which became the recommended drug in the guidelines for HF with reduced ejection fraction (17, 27). However, the PARAGON-HF study presented at the 2019 European Cardiovascular Congress ESC suggested that the trial and control groups of patients with preserved ejection heart failure (HFpEF) did not show a statistical difference, and the effect of ARNI in patients with non-reduced ejection fraction HF remains controversial (28). So, patients with reduced ejection fraction HF will be excluded in this trial to avoid ethical conflicts.

The anti-arrhythmic efficacy of sacubitril/valsartan is also being noticed by researchers. ARNI can reduce atrial arrhythmic events in HF with reduced ejection fraction. It is shown that a trend toward a reduction in the frequency of atrial arrhythmia events after nine months of taking ARNI, suggesting a possible link between ARNI and atrial arrhythmia (24). Sacubitril acts synergistically by modulating the natriuretic peptide system, thus allowing ARNI to make greater contributions in reversing atrial remodeling. So, it is reasonable to postulate that the use of ARNI after catheter ablation of atrial fibrillation reverses left atrial electrical remodeling and positively contributes to sinus rhythm maintenance after catheter ablation. Okutucu from Turkey found that the *P*-wave dispersion and maximum *P*-wave time frame improved by using ARNI in 28 patients with reduced ejection fraction HF, suggesting that ARNI may have reversed left atrial remodeling in patients with reduced ejection fraction HF (29). Also, DeVecchis published a retrospective cohort study showing that patients with HF combined with atrial fibrillation had an increasing peak atrial longitudinal strain (PALS) after ARNI treatment, which also reduced the risk of atrial fibrillation recurrence in such patients (30). Li from China found that rabbits with heart failure experienced prolongation of the effective atrial and ventricular induction periods and altered calcium channel proteins in a rabbit model of AF. These results suggest that ARNI improves atrial electrical and structural remodeling in AF (31). We surmise that Sacubitril/valsartan can reverse cardiac remodeling and reduce myocardial fibrosis, so as to reduce the recurrence of AF and the incidence of adverse cardiovascular outcomes in order to improve the prognosis of catheter ablation.

ARNI also has been approved for the therapy of hypertension and has a strong anti-hypertensive effect. In clinical practice, hypertension is a high-risk factor for HF in atrial fibrillation patients. The anti-hypertensive function of ARNI may contribute to reduce the incidence of adverse cardiovascular events after catheter ablation of AF (32).

In conclusion, ARNI may reduce the recurrence of AF and the incidence of cardiovascular adverse events due to reduction of myocardial fibrosis, reversing cardiac remodeling and controlling blood pressure. However, there lack multi-center randomized controlled clinical trials showing the effect of ARNI in patients with persistent AF after catheter ablation. Therefore, we will confirm the effect of ARNI on the prognosis of patients with persistent AF after catheter ablation through a RCT by evaluating sinus rhythm maintenance rate and incidence of MACE in patients. The results of this trial will further explore the application scope of ARNI and the possibility of improving prognosis after catheter ablation.

### Limitations

Firstly, this is an open-label clinical trial and the results can not represent all AF patients. Secondly, lack of continuous ECG monitoring (such as a handheld smartphone ECG device) at patient follow-up allows for the possibility that some atrial arrhythmias are not monitored, and the chance of false negatives is increased.

### Trial status

The recruitment date is from 2022/08/30 To 2025/08/30. The trial is currently recruiting patients.

## Data Availability

The datasets used and/or analyzed during the current study are available from the corresponding author on reasonable request.
